# *catena*-Poly[[tris[silver(I)-μ-4,4′-bi­pyridine-κ^2^ *N*:*N*′]] tris­(perchlorate) di­hydrate]

**DOI:** 10.1107/S1600536811040153

**Published:** 2011-10-08

**Authors:** Xiao-Ming Hu, Fu-An Li

**Affiliations:** a College of Chemistry and Chemical Engineering, Pingdingshan University, Pingdingshan 467000, Henan, People’s Republic of China

## Abstract

In the title compound, {[Ag_3_(C_10_H_8_N_2_)_3_](ClO_4_)_3_·2H_2_O}_*n*_, one of the Ag^I^ ions, one of the 4,4′-bipyridine (bipy) ligands and one of the perchlorate anions are each situated on a twofold rotation axis. Each Ag^I^ ion is coordinated by two N atoms from two bridging bipy ligands, forming chains along [101]. π–π inter­actions between the pyridine rings [centroid–centroid distances = 3.638 (8) and 3.688 (8) Å] connect the chains. Inter­molecular O—H⋯O hydrogen bonds link the uncoord­inated water mol­ecules and the perchlorate anions.

## Related literature

For background to the network topologies and applications of coordination polymers, see: Du *et al.* (2007 ▶); Hu *et al.* (2003 ▶); Lou *et al.* (2005 ▶); Maspoch *et al.* (2007 ▶); Ockwig *et al.* (2005 ▶); Xiao *et al.* (2006 ▶). For O—H⋯O hydrogen bonds, see: Desiraju (2004 ▶). For π–π inter­actions, see: Zang *et al.* (2010 ▶).
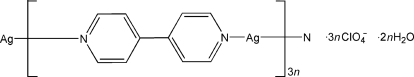


## Experimental

### 

#### Crystal data

[Ag_3_(C_10_H_8_N_2_)_3_](ClO_4_)_3_·2H_2_O
*M* *_r_* = 1126.54
Monoclinic, 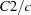

*a* = 21.259 (2) Å
*b* = 15.7647 (17) Å
*c* = 20.949 (3) Å
β = 148.768 (5)°
*V* = 3640.4 (9) Å^3^
*Z* = 4
Mo *K*α radiation
μ = 1.90 mm^−1^
*T* = 296 K
0.21 × 0.20 × 0.19 mm

#### Data collection

Bruker APEXII CCD diffractometer
Absorption correction: multi-scan (*SADABS*; Bruker, 2001 ▶) *T*_min_ = 0.692, *T*_max_ = 0.715
7095 measured reflections
3145 independent reflections
2229 reflections with *I* > 2σ(*I*)
*R*_int_ = 0.037

#### Refinement

*R*[*F* ^2^ > 2σ(*F* ^2^)] = 0.062
*wR*(*F* ^2^) = 0.170
*S* = 1.01
3145 reflections
254 parameters
H-atom parameters constrained
Δρ_max_ = 1.87 e Å^−3^
Δρ_min_ = −1.07 e Å^−3^

### 

Data collection: *APEX2* (Bruker, 2007 ▶); cell refinement: *SAINT* (Bruker, 2007 ▶); data reduction: *SAINT*; program(s) used to solve structure: *SHELXS97* (Sheldrick, 2008 ▶); program(s) used to refine structure: *SHELXL97* (Sheldrick, 2008 ▶); molecular graphics: *DIAMOND* (Brandenburg, 1999 ▶); software used to prepare material for publication: *SHELXTL* (Sheldrick, 2008 ▶).

## Supplementary Material

Crystal structure: contains datablock(s) I, global. DOI: 10.1107/S1600536811040153/hy2472sup1.cif

Structure factors: contains datablock(s) I. DOI: 10.1107/S1600536811040153/hy2472Isup2.hkl

Additional supplementary materials:  crystallographic information; 3D view; checkCIF report

## Figures and Tables

**Table 1:** Hydrogen-bond geometry (Å, °)

*D*—H⋯*A*	*D*—H	H⋯*A*	*D*⋯*A*	*D*—H⋯*A*
O1*W*—H1*WB*⋯O4^i^	0.85	2.21	3.060 (15)	173
O1*W*—H1*WA*⋯O3	0.85	2.44	2.99 (3)	123

Symmetry code: (i) 
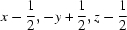
.

**Fig. 1.**
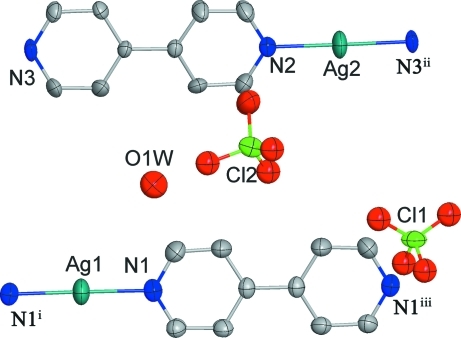
The asymmetric unit of the title compound. Displacement ellipsoids are drawn at the 50% probability level. H atoms are omitted for clarity. [Symmetry codes: (i) -x, y, -z-1/2; (ii) x+1, y, z+1; (iii) -x+1, y, -z+1/2.]

**Fig. 2.**
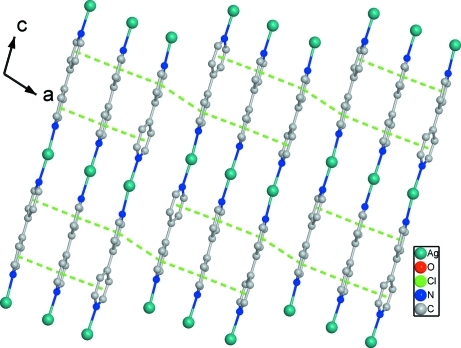
Two-dimensional supramolecular structure in the title compound. Dashed lines indicate π–π interactions.

**Fig. 3.**
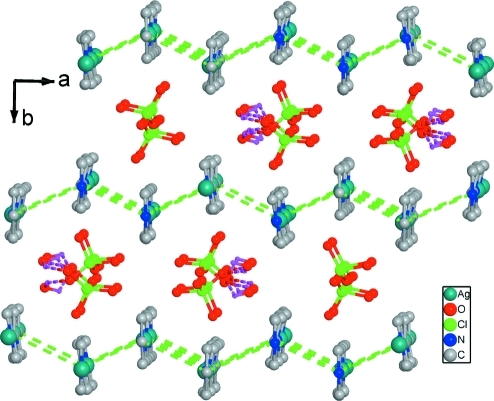
Three-dimensional supramolecular structure of the title compound. Dashed lines indicate π–π interactions and hydrogen bonds.

## supplementary crystallographic information

### Comment

In recent years, supramolecular coordination assemblies have received much attention not only for their variety of architectures but also for potential applications as functional materials (Maspoch *et al.*, 2007; Ockwig *et al.*, 2005). According to literature, 4,4'-bipyridine (bipy) is a good bridging ligand to construct coordination polymers, by which many supramolecular structures have been furnished (Hu *et al.*, 2003; Lou *et al.*, 2005; Xiao *et al.*, 2006). The rational assembly of target metal-organic networks depends on the deliberate designs of ligands with adjustable connectivity and a reasonable choice of metal ions with specific coordination nature. Additionally, the use of auxiliary ligands is also an effective method for the construction of coordination polymers (Du *et al.*, 2007). To further explore the influence of *N*-donor ligands on the properties and construction of coordination polymer, we undertake synthetic and structural studies on an Ag(I) complex based on bipy.

As shown in Fig. 1, the asymmetric unit of the title compound consists of one and a half Ag^I^ ions, one and a half bipy ligands, one water molecule and one and a half perchlorate anions. Each Ag^I^ ion is two-coordinated by two N atoms from two bipy ligands, forming two different one-dimensional chains. Ag2 atom has close contacts with the water molecule and perchlorate anions [Ag2···O1W^i^ = 2.872 (9), Ag2···O1^ii^ = 2.84 (3), Ag···O6^iii^ = 2.91 (1) Å. Symmetry codes: (i) 1/2+x, 1/2-y, 1/2+z; (ii) 3/2-x, 1/2-y, 1-z; (iii) 1-x, -y, 1-z]. The chains are further linked by π–π stacking interactions between different pyridine rings [centroid–centroid distances = 3.638 (8) and 3.688 (8) Å] (Zang *et al.*, 2010), resulting in a two-dimensional supramolecular structure in the *ac* plane (Fig. 2). The two-dimensional supramolecular structures which have positive charge are linked by Ag···O contacts and electrostatic attraction with the perchlorate anions, forming a three-dimensional supramolecular structure. O—H···O hydrogen bonds (Table 1) (Desiraju, 2004) link the lattice water molecules and perchlorate anions (Fig. 3).

### Experimental

A mixture of AgClO_4_.6H_2_O (6.3 mg, 0.02 mmol), 4,4'-bipyridine (3.12 mg, 0.02 mmol) in a 10 ml mixed solution of H_2_O and ethanol (v/v = 1:3) and 5 drops of ammonia was sealed in a stainless-steel reactor with a Teflon liner and heated at 393 K for 72 h. A quantity of colorless single crystals were obtained after the mixture was cooled to room temperature at a rate of 10 K h^-1^.

### Refinement

H atoms on C atoms were generated geometrically and refined as riding atoms, with C—H = 0.93 Å and *U*_iso_(H) = 1.2*U*_eq_(C). The approximate positions of water H atoms were obtained from a difference Fourier map and refined as riding, with O—H = 0.85 Å and *U*_iso_(H) = 1.5*U*_eq_(O). The highest residual electron density was found at 0.05 Å from Ag1 atom and the deepest hole at 0.89 Å from Ag2 atom.

### Crystal data

**Table:**

[Ag_3_(C_10_H_8_N_2_)_3_](ClO_4_)_3_·2H_2_O	*F*(000) = 2216
*M**_r_* = 1126.54	*D*_x_ = 2.056 Mg m^−^^3^
Monoclinic, *C*2/*c*	Mo *K*α radiation, λ = 0.71073 Å
Hall symbol: -C 2yc	Cell parameters from 2263 reflections
*a* = 21.259 (2) Å	θ = 3.1–29.0°
*b* = 15.7647 (17) Å	µ = 1.90 mm^−^^1^
*c* = 20.949 (3) Å	*T* = 296 K
β = 148.768 (5)°	Block, colorless
*V* = 3640.4 (9) Å^3^	0.21 × 0.20 × 0.19 mm
*Z* = 4	

### Data collection

**Table:**

Bruker APEXII CCD diffractometer	3145 independent reflections
Radiation source: fine-focus sealed tube	2229 reflections with *I* > 2σ(*I*)
graphite	*R*_int_ = 0.037
φ and ω scans	θ_max_ = 25.0°, θ_min_ = 3.2°
Absorption correction: multi-scan (*SADABS*; Bruker, 2001)	*h* = −25→25
*T*_min_ = 0.692, *T*_max_ = 0.715	*k* = −12→18
7095 measured reflections	*l* = −23→24

### Refinement

**Table:**

Refinement on *F*^2^	Primary atom site location: structure-invariant direct methods
Least-squares matrix: full	Secondary atom site location: difference Fourier map
*R*[*F*^2^ > 2σ(*F*^2^)] = 0.062	Hydrogen site location: inferred from neighbouring sites
*wR*(*F*^2^) = 0.170	H-atom parameters constrained
*S* = 1.01	*w* = 1/[σ^2^(*F*_o_^2^) + (0.0911*P*)^2^] where *P* = (*F*_o_^2^ + 2*F*_c_^2^)/3
3145 reflections	(Δ/σ)_max_ = 0.001
254 parameters	Δρ_max_ = 1.87 e Å^−^^3^
0 restraints	Δρ_min_ = −1.07 e Å^−^^3^

### Fractional atomic coordinates and isotropic or equivalent isotropic displacement parameters (Å^2^)

**Table:**

	*x*	*y*	*z*	*U*_iso_*/*U*_eq_	
Ag1	0.0000	0.45375 (5)	−0.2500	0.0566 (3)	
Ag2	0.59795 (4)	0.05250 (4)	0.68999 (4)	0.0558 (3)	
O1	0.9612 (11)	0.3396 (11)	0.2576 (10)	0.320 (11)	
O2	1.0878 (12)	0.2447 (7)	0.3549 (11)	0.225 (6)	
O3	0.2999 (8)	0.2382 (7)	0.1888 (9)	0.183 (5)	
O4	0.4471 (17)	0.2642 (6)	0.3825 (14)	0.279 (8)	
O5	0.4822 (10)	0.2063 (8)	0.3197 (13)	0.240 (6)	
O6	0.3993 (6)	0.1309 (6)	0.3237 (6)	0.147 (3)	
O1W	0.1272 (4)	0.2966 (4)	0.1401 (5)	0.0789 (16)	
H1WB	0.0798	0.2751	0.0713	0.118*	
H1WA	0.2000	0.2788	0.2016	0.118*	
N1	0.1878 (5)	0.4546 (3)	−0.0596 (5)	0.0465 (14)	
N2	0.4098 (5)	0.0531 (3)	0.4983 (5)	0.0404 (12)	
N3	−0.2117 (4)	0.0492 (3)	−0.1224 (5)	0.0410 (13)	
C1	0.2488 (6)	0.5264 (4)	−0.0009 (6)	0.0390 (14)	
H1	0.2081	0.5774	−0.0424	0.047*	
C2	0.3715 (6)	0.5285 (4)	0.1205 (6)	0.0386 (14)	
H2	0.4115	0.5803	0.1582	0.046*	
C3	0.4347 (5)	0.4539 (4)	0.1856 (5)	0.0318 (13)	
C4	0.3680 (6)	0.3788 (4)	0.1220 (7)	0.0522 (17)	
H4	0.4053	0.3266	0.1612	0.063*	
C5	0.2469 (6)	0.3825 (5)	0.0010 (6)	0.0560 (19)	
H5	0.2044	0.3319	−0.0402	0.067*	
C6	0.3549 (6)	−0.0171 (4)	0.4321 (6)	0.0480 (16)	
H6	0.3989	−0.0675	0.4706	0.058*	
C7	0.2379 (5)	−0.0197 (4)	0.3112 (5)	0.0459 (16)	
H7	0.2051	−0.0703	0.2679	0.055*	
C8	0.1676 (5)	0.0518 (3)	0.2521 (5)	0.0295 (13)	
C9	0.2230 (6)	0.1252 (4)	0.3215 (6)	0.0461 (16)	
H9	0.1792	0.1755	0.2862	0.055*	
C10	0.3441 (6)	0.1232 (4)	0.4441 (6)	0.0524 (17)	
H10	0.3805	0.1729	0.4900	0.063*	
C11	0.0359 (5)	0.0509 (3)	0.1213 (5)	0.0279 (12)	
C12	−0.0267 (5)	−0.0238 (4)	0.0584 (5)	0.0318 (13)	
H12	0.0135	−0.0754	0.0971	0.038*	
C13	−0.1494 (5)	−0.0225 (4)	−0.0625 (5)	0.0382 (14)	
H13	−0.1898	−0.0737	−0.1032	0.046*	
C14	−0.1510 (6)	0.1215 (4)	−0.0627 (6)	0.0534 (18)	
H14	−0.1926	0.1725	−0.1033	0.064*	
C15	−0.0293 (5)	0.1237 (4)	0.0570 (6)	0.0490 (17)	
H15	0.0094	0.1759	0.0948	0.059*	
Cl1	1.0000	0.28764 (14)	0.2500	0.0493 (6)	
Cl2	0.41235 (16)	0.21081 (11)	0.30907 (16)	0.0539 (5)	

### Atomic displacement parameters (Å^2^)

**Table:**

	*U*^11^	*U*^22^	*U*^33^	*U*^12^	*U*^13^	*U*^23^
Ag1	0.0280 (4)	0.0644 (6)	0.0340 (5)	0.000	0.0197 (4)	0.000
Ag2	0.0251 (3)	0.0706 (5)	0.0256 (3)	0.0006 (2)	0.0144 (3)	0.0007 (2)
O1	0.233 (13)	0.47 (2)	0.132 (8)	0.233 (16)	0.137 (10)	0.076 (12)
O2	0.310 (15)	0.146 (9)	0.195 (10)	0.143 (9)	0.212 (11)	0.116 (7)
O3	0.136 (6)	0.223 (12)	0.165 (8)	0.107 (8)	0.125 (7)	0.141 (8)
O4	0.51 (2)	0.090 (7)	0.279 (15)	−0.072 (11)	0.343 (19)	−0.066 (9)
O5	0.218 (9)	0.225 (13)	0.388 (15)	0.090 (10)	0.277 (12)	0.107 (12)
O6	0.101 (5)	0.110 (7)	0.090 (5)	−0.024 (5)	0.060 (5)	0.013 (4)
O1W	0.069 (3)	0.069 (4)	0.076 (4)	0.002 (3)	0.058 (3)	0.003 (3)
N1	0.033 (3)	0.048 (4)	0.035 (3)	0.002 (2)	0.025 (3)	0.002 (3)
N2	0.027 (3)	0.040 (3)	0.032 (3)	0.000 (2)	0.022 (3)	0.003 (2)
N3	0.017 (2)	0.051 (4)	0.020 (3)	0.001 (2)	0.010 (2)	0.006 (2)
C1	0.037 (3)	0.034 (3)	0.039 (3)	−0.001 (3)	0.032 (3)	−0.003 (3)
C2	0.042 (3)	0.030 (3)	0.038 (3)	0.001 (3)	0.033 (3)	−0.001 (3)
C3	0.027 (3)	0.041 (4)	0.030 (3)	0.000 (2)	0.024 (3)	0.000 (3)
C4	0.040 (3)	0.031 (4)	0.051 (4)	0.000 (3)	0.033 (3)	0.001 (3)
C5	0.043 (4)	0.042 (4)	0.043 (4)	−0.006 (4)	0.030 (4)	−0.007 (4)
C6	0.035 (3)	0.043 (4)	0.034 (3)	0.012 (3)	0.024 (3)	0.004 (3)
C7	0.032 (3)	0.043 (4)	0.027 (3)	0.003 (3)	0.019 (3)	−0.006 (3)
C8	0.023 (3)	0.033 (3)	0.023 (3)	−0.002 (2)	0.018 (3)	0.000 (2)
C9	0.038 (3)	0.023 (3)	0.039 (4)	−0.007 (3)	0.027 (3)	−0.001 (3)
C10	0.034 (3)	0.037 (4)	0.037 (4)	−0.009 (3)	0.022 (3)	−0.006 (3)
C11	0.022 (3)	0.028 (3)	0.019 (3)	−0.004 (2)	0.015 (3)	−0.002 (2)
C12	0.024 (3)	0.031 (3)	0.029 (3)	0.005 (2)	0.020 (3)	0.003 (3)
C13	0.026 (3)	0.041 (3)	0.022 (3)	−0.004 (3)	0.017 (3)	−0.007 (3)
C14	0.029 (3)	0.035 (4)	0.027 (3)	0.004 (3)	0.013 (3)	0.010 (3)
C15	0.036 (3)	0.031 (4)	0.029 (3)	−0.004 (3)	0.020 (3)	0.005 (3)
Cl1	0.0637 (15)	0.0265 (11)	0.0578 (15)	0.000	0.0520 (14)	0.000
Cl2	0.0567 (11)	0.0389 (9)	0.0568 (11)	0.0052 (8)	0.0470 (10)	0.0060 (8)

### Geometric parameters (Å, °)

**Table:**

Ag1—N1	2.149 (6)	C3—C4	1.400 (8)
Ag1—N1^i^	2.149 (6)	C3—C3^iii^	1.475 (12)
Ag2—N3^ii^	2.151 (5)	C4—C5	1.378 (10)
Ag2—N2	2.158 (6)	C4—H4	0.9300
O1—Cl1	1.27 (3)	C5—H5	0.9300
O2—Cl1	1.325 (9)	C6—C7	1.353 (8)
O3—Cl2	1.396 (8)	C6—H6	0.9300
O4—Cl2	1.295 (10)	C7—C8	1.368 (8)
O5—Cl2	1.30 (3)	C7—H7	0.9300
O6—Cl2	1.379 (8)	C8—C9	1.383 (8)
O1W—H1WB	0.8500	C8—C11	1.492 (8)
O1W—H1WA	0.8500	C9—C10	1.385 (9)
N1—C1	1.323 (8)	C9—H9	0.9300
N1—C5	1.324 (9)	C10—H10	0.9300
N2—C6	1.320 (8)	C11—C15	1.364 (8)
N2—C10	1.323 (8)	C11—C12	1.377 (8)
N3—C13	1.328 (8)	C12—C13	1.386 (7)
N3—C14	1.329 (8)	C12—H12	0.9300
C1—C2	1.389 (9)	C13—H13	0.9300
C1—H1	0.9300	C14—C15	1.374 (8)
C2—C3	1.383 (8)	C14—H14	0.9300
C2—H2	0.9300	C15—H15	0.9300
			
N1—Ag1—N1^i^	179.3 (3)	C9—C8—C11	120.8 (5)
N3^ii^—Ag2—N2	176.36 (19)	C8—C9—C10	119.5 (6)
H1WB—O1W—H1WA	115.0	C8—C9—H9	120.3
C1—N1—C5	118.1 (6)	C10—C9—H9	120.3
C1—N1—Ag1	121.3 (4)	N2—C10—C9	122.4 (6)
C5—N1—Ag1	120.5 (5)	N2—C10—H10	118.8
C6—N2—C10	117.6 (6)	C9—C10—H10	118.8
C6—N2—Ag2	121.2 (4)	C15—C11—C12	116.1 (6)
C10—N2—Ag2	121.1 (4)	C15—C11—C8	122.1 (5)
C13—N3—C14	117.3 (5)	C12—C11—C8	121.7 (5)
C13—N3—Ag2^iv^	123.0 (4)	C11—C12—C13	120.3 (6)
C14—N3—Ag2^iv^	119.7 (4)	C11—C12—H12	119.9
N1—C1—C2	122.4 (6)	C13—C12—H12	119.9
N1—C1—H1	118.8	N3—C13—C12	122.5 (6)
C2—C1—H1	118.8	N3—C13—H13	118.7
C3—C2—C1	120.4 (6)	C12—C13—H13	118.7
C3—C2—H2	119.8	N3—C14—C15	122.5 (6)
C1—C2—H2	119.8	N3—C14—H14	118.8
C2—C3—C4	116.2 (6)	C15—C14—H14	118.8
C2—C3—C3^iii^	121.7 (4)	C11—C15—C14	121.2 (6)
C4—C3—C3^iii^	122.1 (4)	C11—C15—H15	119.4
C5—C4—C3	119.6 (6)	C14—C15—H15	119.4
C5—C4—H4	120.2	O1^v^—Cl1—O1	99.3 (17)
C3—C4—H4	120.2	O1^v^—Cl1—O2^v^	105.5 (7)
N1—C5—C4	123.3 (6)	O1—Cl1—O2^v^	113.2 (9)
N1—C5—H5	118.4	O1^v^—Cl1—O2	113.2 (9)
C4—C5—H5	118.4	O1—Cl1—O2	105.5 (7)
N2—C6—C7	123.4 (6)	O2^v^—Cl1—O2	118.6 (12)
N2—C6—H6	118.3	O4—Cl2—O5	115.0 (10)
C7—C6—H6	118.3	O4—Cl2—O6	110.4 (7)
C6—C7—C8	120.5 (6)	O5—Cl2—O6	109.2 (6)
C6—C7—H7	119.8	O4—Cl2—O3	107.5 (9)
C8—C7—H7	119.8	O5—Cl2—O3	105.6 (7)
C7—C8—C9	116.7 (6)	O6—Cl2—O3	108.9 (5)
C7—C8—C11	122.5 (5)		
			
C5—N1—C1—C2	0.5 (10)	C6—N2—C10—C9	−2.1 (10)
Ag1—N1—C1—C2	−177.1 (4)	Ag2—N2—C10—C9	−179.5 (5)
N1—C1—C2—C3	−0.8 (9)	C8—C9—C10—N2	−0.1 (10)
C1—C2—C3—C4	0.2 (9)	C7—C8—C11—C15	−169.4 (6)
C1—C2—C3—C3^iii^	179.6 (6)	C9—C8—C11—C15	14.1 (9)
C2—C3—C4—C5	0.8 (10)	C7—C8—C11—C12	10.0 (9)
C3^iii^—C3—C4—C5	−178.7 (7)	C9—C8—C11—C12	−166.5 (5)
C1—N1—C5—C4	0.5 (11)	C15—C11—C12—C13	−1.4 (8)
Ag1—N1—C5—C4	178.1 (6)	C8—C11—C12—C13	179.1 (5)
C3—C4—C5—N1	−1.1 (11)	C14—N3—C13—C12	1.3 (9)
C10—N2—C6—C7	4.0 (10)	Ag2^iv^—N3—C13—C12	179.9 (4)
Ag2—N2—C6—C7	−178.7 (5)	C11—C12—C13—N3	−0.1 (9)
N2—C6—C7—C8	−3.6 (11)	C13—N3—C14—C15	−1.0 (10)
C6—C7—C8—C9	1.2 (9)	Ag2^iv^—N3—C14—C15	−179.7 (5)
C6—C7—C8—C11	−175.4 (6)	C12—C11—C15—C14	1.7 (9)
C7—C8—C9—C10	0.5 (9)	C8—C11—C15—C14	−178.8 (6)
C11—C8—C9—C10	177.2 (6)	N3—C14—C15—C11	−0.5 (11)

Symmetry codes: (i) −*x*, *y*, −*z*−1/2; (ii) *x*+1, *y*, *z*+1; (iii) −*x*+1, *y*, −*z*+1/2; (iv) *x*−1, *y*, *z*−1; (v) −*x*+2, *y*, −*z*+1/2.

### Hydrogen-bond geometry (Å, °)

**Table:**

*D*—H···*A*	*D*—H	H···*A*	*D*···*A*	*D*—H···*A*
O1W—H1WB···O4^vi^	0.85	2.21	3.060 (15)	173
O1W—H1WA···O3	0.85	2.44	2.99 (3)	123

Symmetry codes: (vi) *x*−1/2, −*y*+1/2, *z*−1/2.

## References

[bb1] Brandenburg, K. (1999). *DIAMOND* Crystal Impact GbR, Bonn, Germany.

[bb2] Bruker (2001). *SADABS* Bruker AXS Inc., Madison, Wisconsin, USA.

[bb3] Bruker (2007). *APEX2* and *SAINT* Bruker AXS Inc., Madison, Wisconsin, USA.

[bb4] Desiraju, G. R. (2004). *Hydrogen Bonding. Encyclopedia of Supramolecular Chemistry*, edited by J. L. Atwood & J. W. Steed, pp. 658–665. New York: Marcel Dekker Inc.

[bb5] Du, M., Jiang, X.-J. & Zhao, X.-J. (2007). *Inorg. Chem.* **46**, 3984–3995. 10.1021/ic062098+ 17432846

[bb6] Hu, D.-H., Huang, W., Gou, S.-H., Fang, J.-L. & Fun, H.-K. (2003). *Polyhedron*, **22**, 2661–2667.

[bb7] Lou, B.-Y., Wang, R.-H., Yuan, D.-Q., Wu, B.-L., Jiang, F.-L. & Hong, M.-C. (2005). *Inorg. Chem. Commun.* **8**, 971–974.

[bb8] Maspoch, D., Ruiz-Molina, D. & Veciana, J. (2007). *Chem. Soc. Rev.* **36**, 770–818. 10.1039/b501600m 17471401

[bb9] Ockwig, N. W., Delgado-Friedrichs, O., O’Keefee, M. & Yaghi, O. M. (2005). *Acc. Chem. Res.* **38**, 176–182. 10.1021/ar020022l 15766236

[bb10] Sheldrick, G. M. (2008). *Acta Cryst.* A**64**, 112–122. 10.1107/S0108767307043930 18156677

[bb11] Xiao, D.-R., Wang, E.-B., An, H.-Y., Li, Y.-G., Su, Z.-M. & Sun, C.-Y. (2006). *Chem. Eur. J.* **12**, 6528–6541. 10.1002/chem.200501308 16773662

[bb12] Zang, S.-Q., Liang, R., Fan, Y.-J., Hou, H.-W. & Mak, T. C. W. (2010). *Dalton Trans.* **39**, 8022–8032. 10.1039/c0dt00374c 20657949

